# Secreted Frizzled-Related Protein 2 Is Associated with Disease Progression and Poor Prognosis in Breast Cancer

**DOI:** 10.1155/2019/6149381

**Published:** 2019-03-03

**Authors:** Chumei Huang, Zhuangjian Ye, Jianxin Wan, Jianbo Liang, Min Liu, Xiangdong Xu, Laisheng Li

**Affiliations:** ^1^Department of Laboratory Medicine, The First Affiliated Hospital of Sun Yat-sen University, Guangzhou 510080, China; ^2^Department of Thyroid & Breast Surgery, The First Affiliated Hospital of Sun Yat-sen University, Guangzhou 510080, China

## Abstract

**Purpose:**

Secreted frizzled-related protein 2 (sFRP2) is a secreted protein associated with cancer drug resistance and metastasis. However, few studies have reported serum sFRP2 levels in breast cancer. We evaluated serum sFRP2 as a potential biomarker for breast cancer.

**Methods:**

Serum sFRP2 concentrations were detected in 274 breast cancer patients along with 147 normal healthy controls by enzyme-linked immunosorbent assay (ELISA). Diagnostic significance was evaluated by area under the curve (AUC) analysis and the Youden index. Prognostic significance was determined by Kaplan-Meier survival method and univariate and multivariate Cox proportional hazard regression model analyses.

**Results:**

Serum sFRP2 was elevated in breast cancer patients compared to normal healthy controls (*P* < 0.001). The sensitivity of sFRP2 in diagnosing breast cancer was 76.9% at a specificity of 76.6%. Elevated serum sFRP2 levels are associated with primary tumor size, TNM stage, and lymph node metastases. The Kaplan-Meier curves showed a significant association of serum sFRP2 with progression-free survival. The multivariate Cox analysis confirmed that high serum sFRP2 was an independent prognostic factor for poor prognosis (HR = 3.89, 95% CI = 1.95-7.68, *P* = 0.001).

**Conclusions:**

In conclusion, serum sFRP2 may serve as a potential biomarker for breast cancer diagnosis and prognostic evaluation.

## 1. Introduction

Breast cancer is one of the most frequently malignant tumors and a major cause of cancer-related death among women worldwide [1], with an estimated 268,600 newly diagnosed cases and approximately 69,500 deaths annually in China [2]. Breast cancer is a heterogeneous disease with four major molecular subtypes: luminal A, luminal B, human epidermal growth factor receptor 2- (HER2-) enriched, and basal-like [3]. Although there have been great advances in cancer screening, diagnosis, and treatment in recent years, breast cancer remains a main public health concern, especially for patients with distant metastasis, which contributed to more than 90% of breast cancer deaths [4]. Searching for novel and effective biomarkers to detect early stage breast cancer will facilitate the adoption of less aggressive treatments and ameliorate prognosis [5]. Various identified biomarkers, including CA153, CEA, and CA125, have been widely used in breast cancer patients but have limited clinical value due to low sensitivity and specificity [6, 7]. Thence, in addition to improve the early diagnosis and treatment of this heterogeneous disease, novel noninvasive biomarkers for early diagnosis and prognosis are urgently needed.

Secreted frizzled-related protein 2 (sFRP2), a member of the Wnt-binding protein family, approximately 30-35 kDa, with homology to the transmembrane Wnt-binding domain of frizzled receptors, is widely generated in many adult tissues, including the heart, lung, pancreas, prostate, kidney, and brain [8, 9]. sFRP2 may play diverse roles in tissue morphogenesis through mediator on Wnt signaling [7, 8]. sFRP2 has recently been found to drive tumorigenesis, tumor metastasis, and drug resistance in several cancers, including melanoma, renal cancer, and breast cancer [10–12]. These studies indicated that sFRP2 could be a potential biomarker of breast cancer. However, to the best of our knowledge, serum sFRP2 has not yet been reported in breast cancer.

In this study, to explore the diagnostic and prognostic value of serum sFRP2 in breast cancer, we examined serum levels in breast cancer patients by enzyme-linked immunosorbent assay (ELISA) and assessed the association between serum sFRP2 and clinicopathological features.

## 2. Materials and Methods

### 2.1. Patients and Specimens

Samples used in this study were collected at the First Affiliated Hospital of Sun Yat-sen University. Two hundred and seventy-four stage I-III primary breast cancer patients were investigated from January 2004 to January 2009. All breast cancer patients were diagnosed and confirmed independently by two pathologists, who performed pathological examination of specimens coming from biopsies or surgically resected tissues, according to the World Health Organization (WHO) criteria. All the enrolled subjects presented with tumors that were restricted to the breast, with no indication of distant metastasis or skin involvement at presentation. Patients who had previous malignancy or received neoadjuvant chemotherapy or preoperative radiation therapy prior to surgical operation were excluded from the study. All the patients received standard surgical treatment attached with endocrine therapy, adjuvant chemotherapy, and radiotherapy under the guidelines of National Comprehensive Cancer Network. All clinicopathological data, including age, histology, tumor size, lymph node status, and follow-up data, were collected from medical records. The tumor stage was determined according to the criteria for breast cancer of the American Joint Committee on Cancer. The control group contained 147 age-matched healthy volunteers who performed physical examination at the Department of Physical Health Examination of the First Affiliated Hospital of Sun Yat-sen University. All enrolled subjects were unrelated ethnic Han Chinese women. All subjects with breast cancer were followed up at intervals of three to 12 months (every three months for the first two years, then every six months for three years, and yearly thereafter) until June 2016. Progression-free survival (DFS) was defined as the date from diagnosis to the date the patient lives with the disease but it does not get worse.

The research protocol was reviewed and approved by the Human Research Ethics Committee of the First Affiliated Hospital of Sun Yat-sen University in accordance with the principles of the Declaration of Helsinki. Each participant provided informed consent for participation in the study at the first visit.

### 2.2. Serum Collection and sFRP2 Measurement

All serum samples were collected directly from breast cancer patients at the time of first visit and stored immediately at -80°C. The serum concentration of human sFRP2 was measured using a commercially available sandwich ELISA kit (Elabscience Inc., Wuhan, China) with a detection range of 1.25-80 ng/mL. Measurements were performed completely blinded to the clinical information strictly following manufacturer's instructions, and quality control was guaranteed. The optical density (OD value) was determined at 450 nm by SpectraMax M5 Microplate Reader (Molecular Devices, USA).

### 2.3. Statistical Analyses

Statistical analyses were carried out using SPSS (Version 13.0, SPSS Inc., Chicago, IL, USA) and GraphPad Prism (Version 5.0, GraphPad Software Inc., San Diego, CA, USA). All variables with normal distribution were expressed as mean ± standard deviation (SD). Mann-Whitney *U* test or the Wilcoxon *t*-test was applied to calculate differences between groups. Spearman's rank correlation test was used to evaluate the correlations between groups. Receiver operating characteristic (ROC) curves were applied to calculate the diagnostic value of the biomarkers. The cut-off level for sFRP2 quantification was calculated by the Youden index. Survival curves were determined by the Kaplan-Meier method and analyzed by the log-rank test. Cox proportional hazard regression model was applied for univariate and multivariate analyses of correlation between clinicopathological variables and PFS. In all cases, statistical tests were two-sided, and *P* values less than 0.05 were considered statistically significant.

## 3. Results

### 3.1. Clinicopathological Characteristics of Patients

The study recruited a total of 274 patients who were treated for breast cancer at the First Affiliated Hospital of Sun Yat-sen University and 147 normal healthy controls. The clinicopathological characteristics of the study subjects are displayed in Table 1. The median age of the breast cancer patients was 46 years. Among all cases, 173 (63.2%) patients had a large tumor size (>2 cm), 136 (49.6%) patients had lymph node metastases, 172 (62.8%) patients had estrogen receptor- (ER-) positive tumors, 149 (54.4%) patients had progesterone receptor- (PR-) positive tumors, and 71 (25.8%) patients had HER2-positive tumors. Clinical treatment of all patients was according to NCCN guidelines, including surgery, chemotherapy, hormone therapy, radiotherapy, and trastuzumab, with a median follow-up of 91 months (ranging from 8 to 120 months).

### 3.2. Relationship between Serum sFRP2 and Clinicopathological Characteristics of Patients

**Figure 1:**
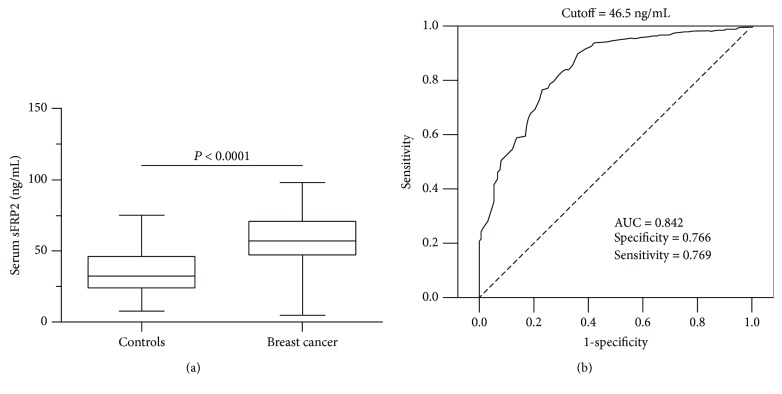
Diagnostic performance of serum sFRP2 in breast cancer patients. (a) Serum sFRP2 levels in breast cancer patients and normal healthy controls. The mean level of serum sFRP2 for breast cancer patients was 58.8 ± 18.2 ng/mL, which is significantly higher than that for healthy controls (34.9 ± 15.5 ng/mL, *P* < 0.0001). (b) ROC analysis of serum sFRP2 levels in distinguishing breast cancer patients from normal healthy controls (AUC = 0.842, 95% CI: 0.803-0.882, *P* < 0.01).

We first measured serum sFRP2 levels in samples by ELISA. As displayed in Table 2, the mean serum concentration of sFRP2 in breast cancer patients was 58.8 ± 18.2 ng/mL, which was obviously higher than the mean serum sFRP2 level in normal healthy controls (Figure 1(a), mean 34.9 ± 15.5 ng/mL, *P* < 0.001). Then, we analyzed the relationship between serum sFRP2 and clinicopathological characteristics of breast cancer patients. As shown in Table 2, serum sFRP2 levels were correlated with the tumor size, TNM stage, and lymph node metastases status (*P* < 0.05). Patients with large primary tumor size, advanced TNM stage, and lymph node metastases showed elevated serum sFRP2 concentrations. There was no relationship between serum sFRP2 and age, menopausal status, histopathological subtype, or ER, PR, or HER2 status (*P* > 0.05).

### 3.3. Serum sFRP2 Acts as a Potential Diagnostic Biomarker for Breast Cancer

To assess the performance of serum sFRP2 as a diagnostic biomarker in distinguishing breast cancer patients from normal healthy controls, receiver operating characteristic/area under the curve (ROC/AUC) was performed (Figure 1(b)). The ROC analysis showed that the AUC was 0.842 (95% CI: 0.803-0.882, *P* < 0.01). The cut-off value was shown to be 46.5 ng/mL, which was determined by the Youden index, and the sensitivity and specificity were 76.6% and 76.9%, respectively. We used logistic models to compare the sensitivity of sFRP2, CEA, and CA153 for the differentiation of breast cancer vs. normal controls at set specificity of approximately 90, 95, and 98% (Table 3). sFRP2 displayed an obviously higher sensitivity, compared with CEA and CA153 for detecting breast cancer. For instance, at a specificity of 95%, sFRP2 had a sensitivity of 30%, higher than CEA (15%), and CA153 (23%). Overall, our data demonstrated that serum sFRP2 shows a similar diagnostic performance for breast cancer patients compared to conventional biomarkers.

### 3.4. Elevated Serum sFRP2 Was Correlated with Poor Prognosis of Patients with Breast Cancer

**Figure 2:**
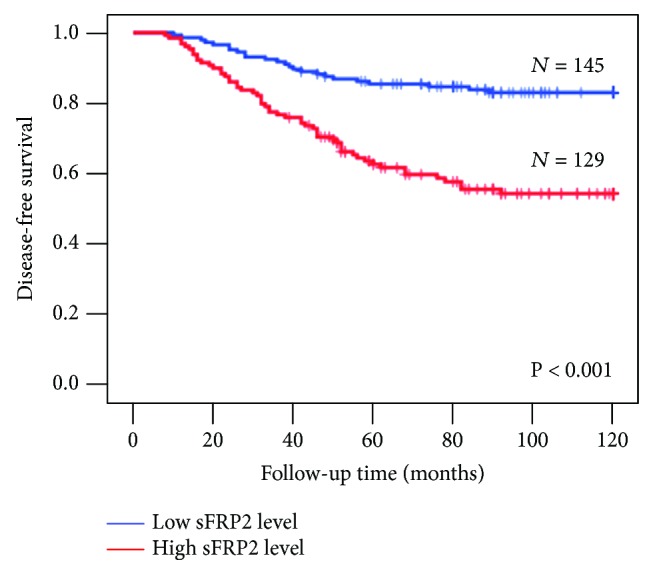
Kaplan-Meier survival curves of breast cancer patients. Progression-free survival rate of breast cancer patients with high (>58 ng/mL) and low (≤58 ng/mL) serum sFRP2 levels.

To further explore the prognostic role of serum sFRP2 in patients with breast cancer, breast cancer patients' outcomes were analyzed by Kaplan-Meier analysis. The breast cancer patients' median concentration of serum sFRP2 (58 ng/mL) was categorized as a threshold to divide the 274 breast cancer patients into two groups: a high serum sFRP2 group (>58 ng/mL, *n* = 129) and a low serum sFRP2 group (≤58 ng/mL, *n* = 145). As shown in Figure 2, high serum sFRP2 group patients received a poor prognosis compared with the low serum sFRP2 group patients. In univariate Cox regression analysis, menopausal status, ER status, tumor size, lymph node status, TNM stage, and serum sFRP2 concentration were significantly correlated with the risk of poor prognosis (*P* < 0.05). No obvious association was displayed between age, PR status, or HER2 status and patients' prognosis (Table 4). In multivariate analysis, menopausal status, ER status, lymph node status, TNM stage, and serum sFRP2 concentration were obviously correlated with the risk of poor prognosis (Table 4, *P* < 0.05). Other clinicopathological characteristics did not show significant association with prognosis.

## 4. Discussion

sFRP2, approximately 300 amino acids in length, belongs to a large family of sFRPs, which are circulating soluble proteins with a highly homologous cysteine-rich domain for cell surface frizzled receptors [13]. sFRP2 is implicated in regulating the Wnt signaling cascade, which plays a vital role in a series of biological processes, including cell proliferation, differentiation, development, cell migration, angiogenesis, oncogenesis, and metastasis [13–16]. Previous studies have revealed that sFRP2 is dysregulated in many types of cancer. However, the contribution of sFRP2 to the biology of cancers remains controversial. For instance, sFRP2 has been implicated in binding to Wnts, thereby preventing Wnt ligands from binding to frizzled receptors and serving as an inhibitor of the Wnt-catenin pathway [17]. Meanwhile, sFRP2 is downregulated by epigenetic promoter hypermethylation in gastric cancer [18], colorectal cancer [19], melanoma [20], and oral squamous cell carcinoma [21], suggesting that sFRP2 could be regarded as a tumor suppressor. In contrast, the expression of sFRP2 is upregulated in renal cancer [12] and breast cancer [10, 14], resulting in canonical Wnt signaling activation and tumorigenesis [13]. In addition, recent studies have revealed that overexpression of sFRP2 can promote the invasive, metastatic, and therapeutic resistance potential of certain types of cancer [11, 16, 22]. These reports suggest that sFRP2 may play diverse roles in cancer. As a secreted protein, serum sFRP2 could be a diagnostic biomarker in certain type of cancers; however, little is known about serum sFRP2 in breast cancer.

In this study, we found that serum sFRP2 concentrations were increased in breast cancer patients compared with normal healthy controls. We also found that serum sFRP2 concentrations were associated with breast cancer tumor size, TNM stage, and lymph node metastasis status. Kaplan-Meier and Cox regression analysis also found that elevated sFRP2 level was associated with a poor prognosis and can be an independent prognostic factor for breast cancer patients. Furthermore, ROC analysis showed that serum sFRP2 had the potential to distinguish breast cancer patients from normal healthy controls with high sensitivity. Because serum sFRP2 can be conveniently measured by the application of a commercial ELISA kit, our initial results demonstrate the potential value of sFRP2 as a diagnostic biomarker for breast cancer.

Metastasis is the main cause of cancer-associated deaths and is involved in cancer cell processes from the primary tumor to translocation and colonization at the secondary site [23]. In order to metastasize, cancer cells must be aggressive and progressive, disseminating from the primary site and surviving and proliferating in the new site. Additionally, the nutrients must be supplied by simple diffuse, extensive angiogenesis to support the expanding tumor mass. Therefore, the tumor microenvironment and angiogenesis are essential for breast cancer progression [24]. Breast cancer-associated fibroblasts, the most abundant cells in the tumor microenvironment, secrete many biologically active factors, including extracellular matrix components, growth factors, cytokines, and proteases to facilitate the initiation, growth, angiogenesis, invasion, and metastasis of cancer [25]. sFRP2, secreted factor that can influence the microenvironment, is produced by human primary fibroblasts. Sun et al. reported that sFRP2 remarkably induces transcription by the nuclear factor-*κ*B (NF-*κ*B) complex, and sFRP2 augments WNT16B signaling to promote advanced malignancies, particularly by achieving therapeutic resistance in the damaged tumor microenvironment [22]. However, which pathway of NF-*κ*B induces transcription of sFRP2 is not unclear. Kaur et al. found that elevated expression of sFRP2 can drive melanoma metastasis and therapy resistance by specifically influencing the tumor microenvironment through activating a multistep signaling cascade [11]. Kaur et al. found that aged fibroblasts secrete sFRP2, which results in a decrease in *β*-catenin and microphthalmia-associated transcription factor (MITF) and ultimately the loss of APE1, rendering the cancer cells more resistant to targeted therapy. Therefore, sFRP2, which is associated with the progression and metastasis of cancer, could be a useful biomarker for its diagnosis and prediction.

Our group aimed to identify a novel serum biomarker for cancer diagnosis [26–28]. Recently, a few groups have independently reported that sFRP2 is highly expressed in breast cancer and associated with tumor progression [10, 14], which led us to evaluate serum sFRP2 as a biomarker for breast cancer diagnosis. We first measured the serum sFRP2 concentration using an ELISA kit in 274 breast cancer patients and 147 normal healthy controls. This showed that serum sFRP2 was elevated in breast cancer patients, and it was associated with the disease progression of cancer. We also found that the mean serum concentration of sFRP2 in TNM stage I+II breast cancer patients was 54.4 ± 16.7 ng/mL, which was obviously higher than the mean serum sFRP2 level in normal healthy controls (34.9 ± 15.5 ng/mL, *P* < 0.001), which implied that sFRP2 could be beneficial in early diagnosis of breast cancer. We then evaluated the diagnosis ability of sFRP2 for breast cancer, and serum sFRP2 displayed significantly higher sensitivity than CEA and CA15.3, with slightly lower specificity. This suggested that serum sFRP2 is a potential diagnostic biomarker for breast cancer. We also found that elevated serum sFRP2 concentrations accompanied poor progression-free survival in breast cancer patients. Contrasting the view that serum sFRP2 acts as a biomarker of poor prognosis, Veeck et al. suggested that low expression of sFRP2 protein in breast cancer tissue was associated with poor prognosis [29].

In conclusion, serum sFRP2 can act as a noninvasive biomarker with high sensitivity for diagnosis and prognosis of breast cancer.

## Figures and Tables

**Table 1:** Clinicopathological characteristics of the study cohort.

Characteristics	No. of patients	(%)	No. of controls	(%)
Age (years)				
≤45	131	47.8	80	54.4
>45	143	52.2	67	45.6
Menopausal status				
Premenopausal	166	60.6	96	65.3
Postmenopausal	108	39.4	51	34.7
Tumor size (cm)				
≤2	79	28.8		
>2	173	63.2		
Undetermined	22	8.0		
Histology				
IDC	213	77.8		
DCIS	59	21.5		
Other	2	0.7		
TNM stage				
I+II	97	35.4		
III	155	56.6		
Undetermined	22	8.0		
Lymph node metastases				
Negative	133	48.6		
Positive	136	49.6		
Unknown	5	1.8		
ER				
Negative	97	35.4		
Positive	172	62.8		
Unknown	5	1.8		
PR				
Negative	120	43.8		
Positive	149	54.4		
Unknown	5	1.8		
HER2				
Negative	198	72.3		
Positive	71	25.8		
Unknown	5	1.9		
Ki67				
≤14%	105	38.3		
>14%	164	59.8		
Unknown	5	1.9		

Abbreviations: IDC: invasive ductal carcinoma; DCIS: ductal carcinoma in situ; ER: estrogen receptor; PR: progesterone receptor; HER2: human epidermal growth factor receptor 2.

**Table 2:** The association of serum sFRP2 levels with clinicopathological characteristics in breast cancer patients.

Characteristics	Number	sFRP2 (mean ± SD, ng/mL)	*P*
Patient group	274	58.8 ± 18.2	**<0.0001**
Control group	147	34.9 ± 15.5	
Age (years)			
≤45	131	57.8 ± 18.3	0.2415
>45	143	60.7 ± 18.7	
Menopausal status			
Premenopausal	166	57.9 ± 18.4	0.1815
Postmenopausal	108	61.4 ± 18.7	
Tumor size (cm)			
≤2	79	54.9 ± 18.5	**0.0118**
>2	173	61.0 ± 18.3	
Histology			
IDC	213	59.8 ± 18.0	0.8597
DCIS	59	57.3 ± 20.3	
TNM stage			
I+II	97	54.4 ± 16.7	**0.0023**
III	155	61.9 ± 19.0	
Lymph node metastases			
Negative	133	53.8 ± 18.2	**<0.0001**
Positive	136	64.6 ± 17.3	
ER			
Negative	97	61.0 ± 18.9	0.1572
Positive	172	58.3 ± 18.3	
PR			
Negative	120	60.3 ± 19.1	0.5532
Positive	149	58.4 ± 18.2	
HER2			
Negative	198	60.2 ± 19.2	0.3460
Positive	71	56.6 ± 16.4	
Ki67			
≤14%	105	55.0 ± 18.2	**0.0253**
>14%	164	62.0 ± 18.3	

**Table 3:** Sensitivity for sFRP2, CEA, and CA15.3 among patients with breast cancer.

Marker	Controls vs. breast cancer patients: sensitivity (%)		
	90% specificity (%)	95% specificity (%)	98% specificity (%)
sFRP2	46	30	21
CEA	32	15	10
CA15.3	41	23	16

**Table 4:** Univariate and multivariate Cox analysis of variables considered for progression-free survival rates of breast cancer patients.

Variables	Category	Univariate analysis			Multivariate analysis		
		HR	95% CI	*P* value	HR	95% CI	*P* value
Age	≤45 Y vs. >45 Y	1.41	0.71-2.74	0.38	1.34	0.69-2.36	0.43
Menopausal status	Post vs. pre	2.13	1.095-3.67	0.02^∗^	2.11	1.42-4.05	0.03^∗^
ER status	Negative vs. positive	1.79	1.06-4.53	0.04^∗^	2.45	1.12-4.61	0.02^∗^
PR status	Negative vs. positive	1.17	0.79-2.12	0.38	1.33	0.72-2.18	0.29
HER2 status	Positive vs. negative	1.95	0.92-4.33	0.09	1.87	0.83-4.04	0.11
Size	>2 cm vs. ≤2 cm	2.91	1.68-5.87	0.003^∗^	1.91	0.91-4.34	0.09
Lymph node status	Positive vs. negative	4.15	1.85-7.03	<0.001^∗^	2.72	1.51-5.95	0.002^∗^
TNM stage	III vs. I+II	2.27	1.17-3.79	0.03^∗^	2.67	1.27-5.72	0.004^∗^
Serum sFRP2	>58.0 vs. ≤58.0 ng/mL	4.14	2.07-8.78	<0.001^∗^	3.89	1.95-7.68	0.001^∗^

Abbreviations: HR: hazard ratio; CI: confidence interval; Y: years; ER status: estrogen receptor status; PR status: progesterone receptor status; HER2 status: human epidermal growth factor receptor 2 status. ^∗^*P* value < 0.05, statistically significant.

## Data Availability

The data used to support the findings of this study are available from the corresponding author upon request.
